# Supplementary material for: Multi-step recognition of potential 5' splice sites by the Saccharomyces cerevisiae U1 snRNP
Source: eLife. 2022 Aug 12;11:e70534. doi: 10.7554/eLife.70534 (PMC9436412; doi:10.7554/eLife.70534)
Supplement: Figure 1—source data 1. [file elife-70534-fig1-data1.docx]

**Figure 1-Source Data 1**

| **Name** | **Sequence^a^** | **ΔG°_unw_ ^b^ (kcal/mol)** |
| --- | --- | --- |
| RNA-10 | 5ʹ - ACU GAA AA**A GGU AAG UAU** AUA UGG ACU GA – **Cy3** | 12.5 |
| RNA-9a | 5ʹ - ACU GAA AA**A GGU AAG UA**A AUA UGG ACU GA – **Cy3** | 12.0 |
| RNA-9b | 5ʹ - ACU GAA AAU **GGU AAG UAU** AUA UGG ACU GA – **Cy3** | 12.4 |
| RNA-8a | 5ʹ - ACU GAA AA**A GGU AAG U**UA AUA UGG ACU GA – **Cy3** | 9.3 |
| RNA-8b | 5ʹ - ACU GAA AAU **GGU AAG UA**A AUA UGG ACU GA – **Cy3** | 11.8 |
| RNA-7a | 5ʹ - ACU GAA AAU **GGU AAG U**UA AUA UGG ACU GA – **Cy3** | 9.1 |
| RNA-7b | 5ʹ - ACU GAA AAU C**GU AAG UA**A AUA UGG ACU GA – **Cy3** | 6.7 |
| RNA-6a | 5ʹ - ACU GAA AAU **GGU AAG** AUA AUA UGG ACU GA – **Cy3** | 8.7 |
| RNA-6b | 5ʹ - ACU GAA AAU C**GU AAG U**UA AUA UGG ACU GA – **Cy3** | 3.9 |
| RNA-5 | 5ʹ - ACU GAA AAU **GGU AA**C AUA AUA UGG ACU GA – **Cy3** | 2.7 |
| RNA-4 | 5ʹ - ACU GAA AAU **GGU A**UC AUA AUA UGG ACU GA – **Cy3** | 3.2 |
| RNA-C | 5ʹ - ACU GAA AAU CCA UUC AUA AUA UGG ACU GA – **Cy3** | ND |
|  |  |  |
| RNA-1+5 | 5ʹ - ACU GAA AAU **GcU AAG U**UA AUA UGG ACU GA – **Cy3** | 0.4 |
| RNA-2+4 | 5ʹ - ACU GAA AAU **Gga AAG U**UA AUA UGG ACU GA – **Cy3** | 3.3 |
| RNA-3+3 | 5ʹ - ACU GAA AAU **GGU uAG U**UA AUA UGG ACU GA – **Cy3** | 4.0 |
| RNA-4+2 | 5ʹ - ACU GAA AAU **GGU AuG U**UA AUA UGG ACU GA – **Cy3** | 4.7 |
| RNA-5+1 | 5ʹ - ACU GAA AAU **GGU Aac U**UA AUA UGG ACU GA – **Cy3** | 2.7 |
|  |  |  |
| RNA-2+7 A(+1) | 5ʹ - ACU GAA AA**A GaU AAG UAU** AUA UGG ACU GA – **Cy3** | 4.4 |
| RNA-2+7 C(+1) | 5ʹ - ACU GAA AA**A GcU AAG UAU** AUA UGG ACU GA – **Cy3** | 4.4 |
| RNA-2+7 U(+1) | 5ʹ - ACU GAA AA**A GuU AAG UAU** AUA UGG ACU GA – **Cy3** | 4.7 |

**^a^** The regions of predicted complementarity to the U1 SSRS are in bold and highlighted. The G(+1) position of the 5ʹ SS (based on RP51A) is underlined.

**^b^** The predicted standard free energy change for duplex unwinding from U1 using DINAMelt at 37^o^C in 1 M NaCl. Note that these free energies were calculated using a uridine-substituted SSRS, see **Methods**.
