# Supplementary material for: Multi-step recognition of potential 5' splice sites by the Saccharomyces cerevisiae U1 snRNP
Source: eLife. 2022 Aug 12;11:e70534. doi: 10.7554/eLife.70534 (PMC9436412; doi:10.7554/eLife.70534)
Supplement: Figure 1—source data 2. [file elife-70534-fig1-data2.docx]

**Figure 1-Source Data 2**

| **RNA^a^** | **N^b^** | **Tau0 (τ_0,_ s)** | **Tau1 (τ_S,_ s)** | **Tau2 (τ_L,_ s)** | **A_L_** | **LLR^b^** |
| --- | --- | --- | --- | --- | --- | --- |
| **RNA-10** | 300 | 155.0 ± 14.0 | 49.2 ± 15.5 | 355.4 ± 115.3 | 0.39 ± 0.12 | *p* < 0.01 |
| **RNA-4+2** | 175 | 55.1 ± 12.5 | 7.8 ± 2.0 | 214.0 ± 87.1 | 0.22 ± 0.05 | *p* < 0.01 |
| **RNA-C** | 26 |  |  |  |  |  |

^a^ Data collected at 1 frame per second

^b^ Number of dwell times combined from replicates for maximum likelihood estimations of single (τ_0_) and double exponential distributions (τ_S_, τ_L_, A_L_). RNA-C had too few events for reliable dwell time analysis

^c^ Results of a loglikelihood ratio test (LLR) comparing the goodness of fit of single and double exponential estimates evaluated at α = 0.01, where *p* < 0.01 favors a double exponential distribution
