# Supplementary material for: Multi-step recognition of potential 5' splice sites by the Saccharomyces cerevisiae U1 snRNP
Source: eLife. 2022 Aug 12;11:e70534. doi: 10.7554/eLife.70534 (PMC9436412; doi:10.7554/eLife.70534)
Supplement: Figure 1—source data 3. [file elife-70534-fig1-data3.docx]

**Figure 1-Source Data 3**

|  | **Model 1** | **Model 2** | **Model 2** |
| --- | --- | --- | --- |
|  |  |  |  |
| Schematic^a^ | 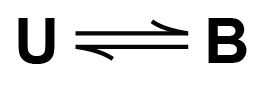 | 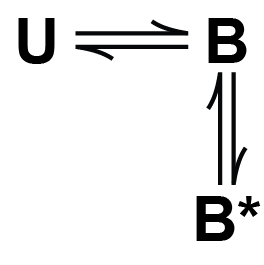 | 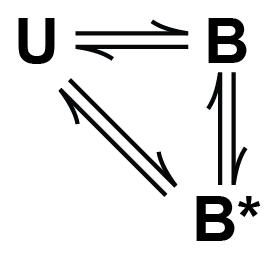 |
| BIC*^b^* | 3.72E+03 ± 2.12E+02 | 3.48E+03 ± 2.47E+02 | 3.54E+03 ± 2.49E+02 |
| ∆⟨BIC⟩^c^ | 249 | 0 | 691 |
|  |  |  |  |
| Rates |  |  |  |
| U 🡪 B (M^-1^, s^-1^) | 2.7E-03 ± 1.6E-04 | 3.2E-03 ± 2.0E-04 | 9.2E-05 ± 9.1E-05 |
| B 🡪 U (s^-1^) | 3.8E-02 ± 2.2E-03 | 1.0E-01 ± 1.2E-02 | 5.1E-04 ± 5.1E-04 |
| B 🡪 B* (s^-1^) |  | 8.4E-03 ± 3.2E-03 | 9.5E-03 ± 1.5E-03 |
| B* 🡪 B (s^-1^) |  | 6.3E-03 ± 1.8E-03 | 1.5E-02 ± 4.4E-03 |
| U 🡪 B* (M^-1^, s^-1^) |  |  | 3.1E-03 ± 2.8E-04 |
| B* 🡪 U (s^-1^) |  |  | 1.3E-01 ± 1.6E-02 |

^a^ U is unbound, B is short-lived bound state, and B* is the long-lived bound state.

^b^ Values reported as mean ± standard deviation of Bayesian information criterion (BIC) scores across a 5-fold resampling of the data

^c^ Average BIC score across resampled data minus the minimum average BIC score.
