# Supplementary figures and images for: Multi-step recognition of potential 5' splice sites by the Saccharomyces cerevisiae U1 snRNP
Source: eLife. 2022 Aug 12;11:e70534. doi: 10.7554/eLife.70534 (PMC9436412; doi:10.7554/eLife.70534)

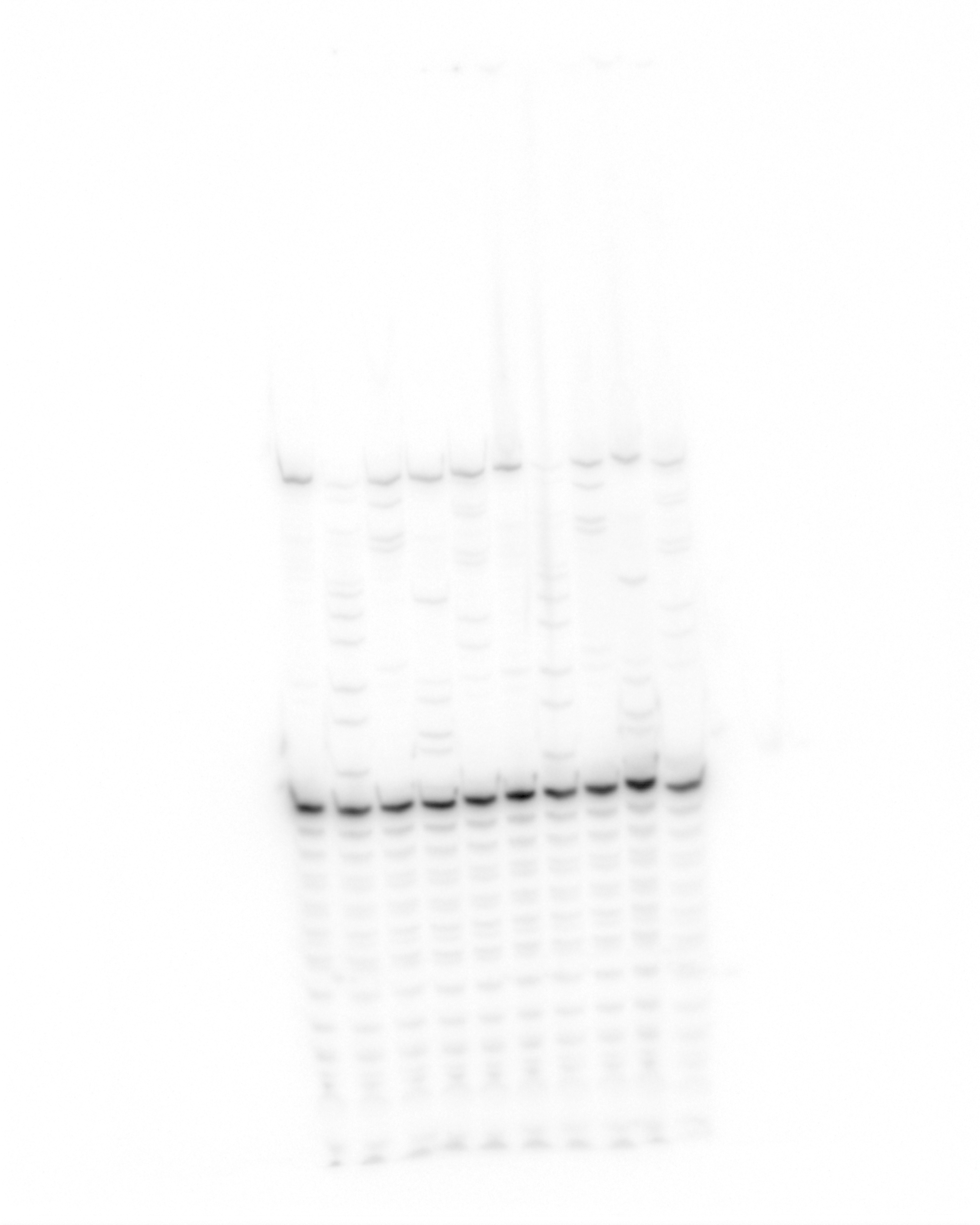

Supplement: Figure 1—figure supplement 2—source data 1. [file elife-70534-fig1-figsupp2-data1.zip › Figure 1-figure supplement 2-source data 1.tiff]

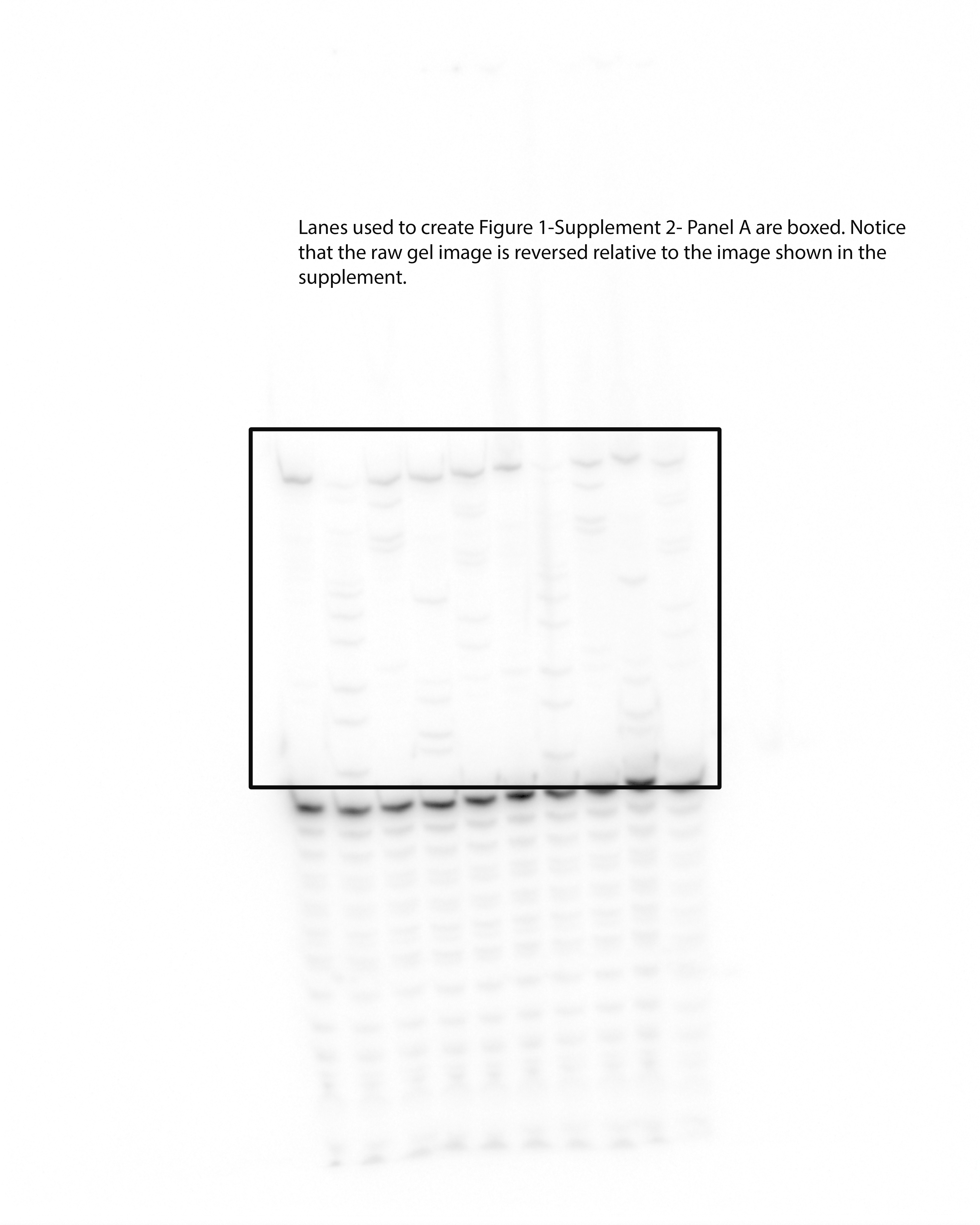

Supplement: Figure 1—figure supplement 2—source data 1. [file elife-70534-fig1-figsupp2-data1.zip › Figure 1-figure supplement 2-source data 1_Marked.jpg]

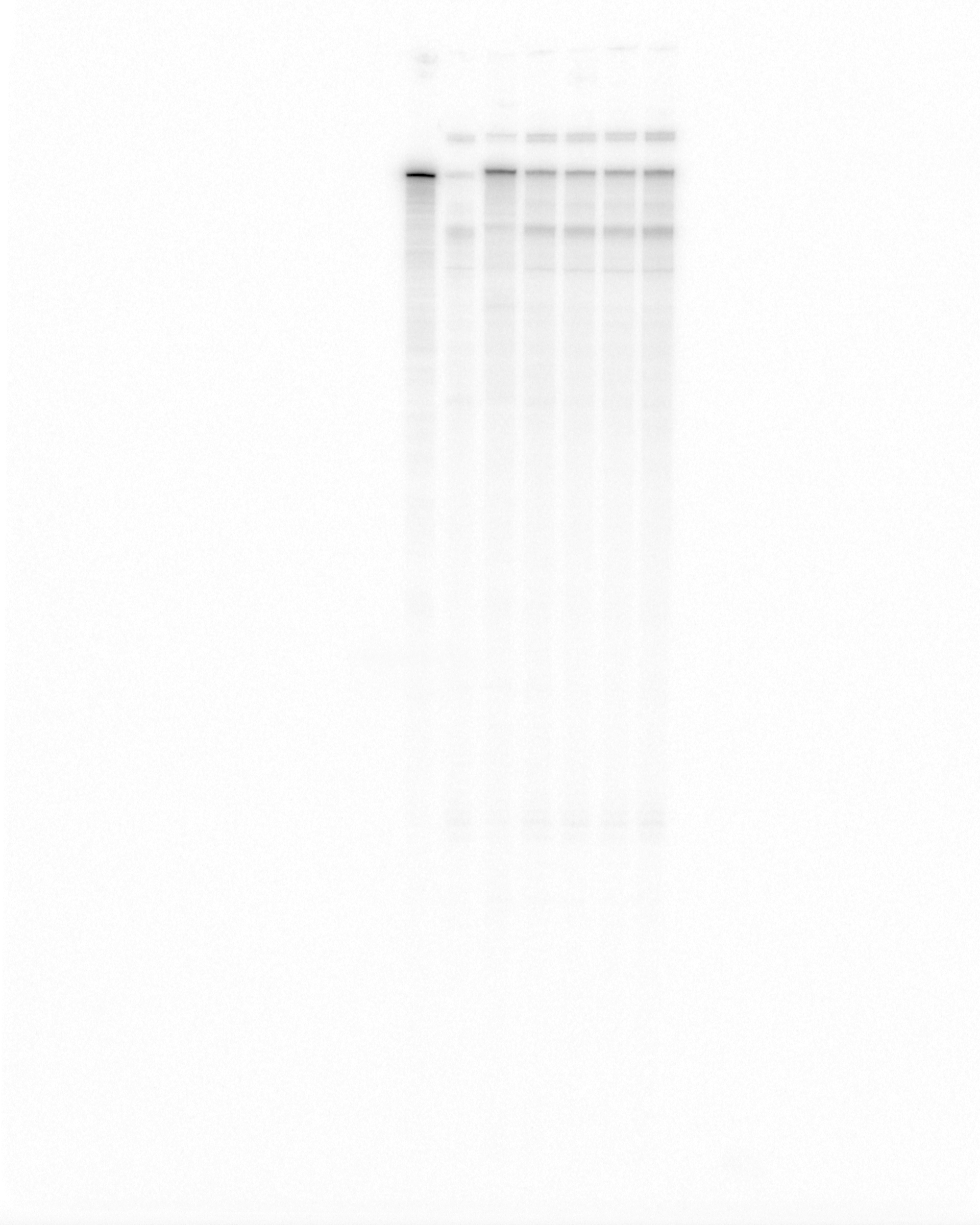

Supplement: Figure 1—figure supplement 2—source data 2. [file elife-70534-fig1-figsupp2-data2.zip › Figure1-figure supplement 2-source data 2.tiff]

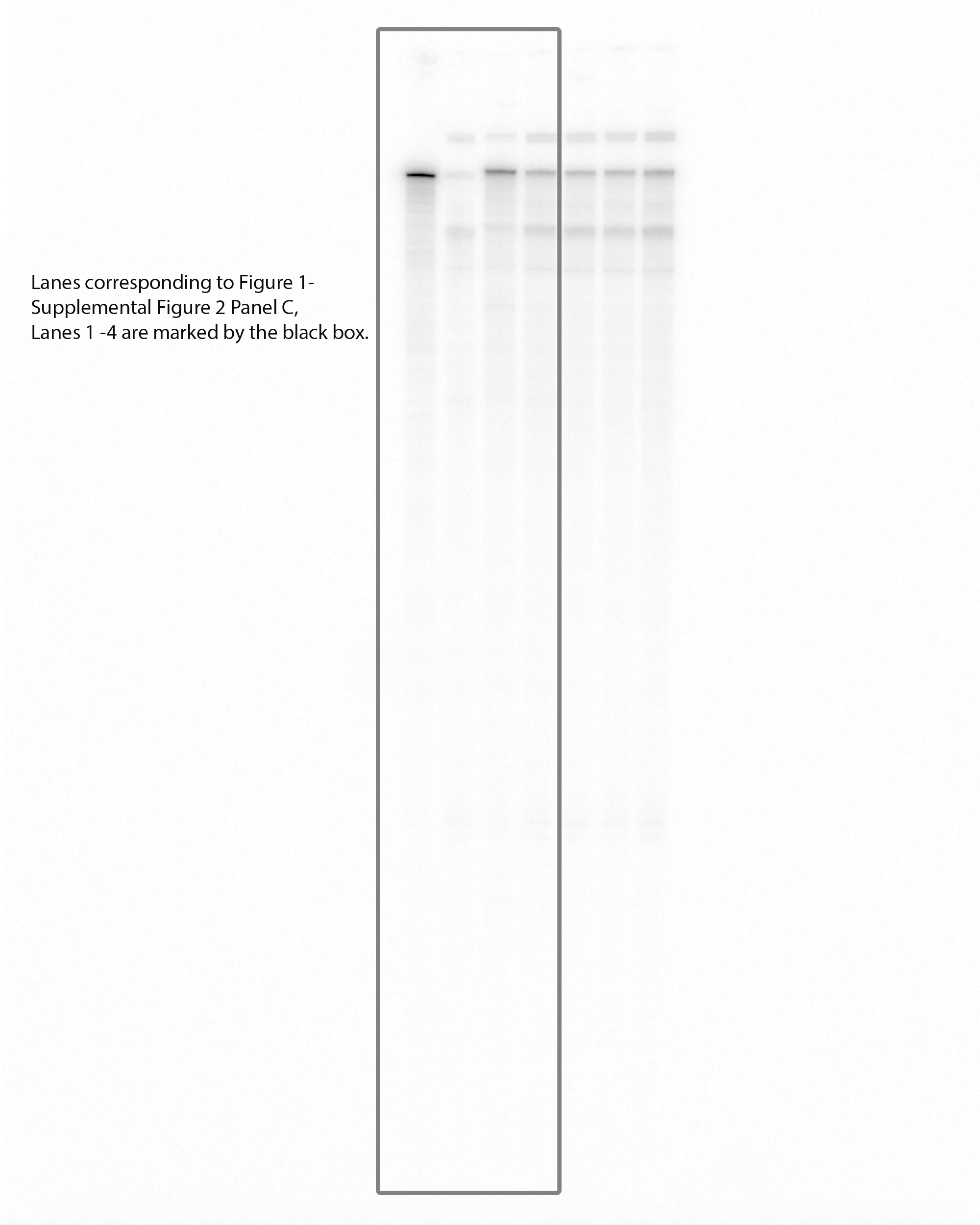

Supplement: Figure 1—figure supplement 2—source data 2. [file elife-70534-fig1-figsupp2-data2.zip › Figure1-figure supplement 2-source data 2_Marked.jpg]
