# Supplementary material for: Multi-step recognition of potential 5' splice sites by the Saccharomyces cerevisiae U1 snRNP
Source: eLife. 2022 Aug 12;11:e70534. doi: 10.7554/eLife.70534 (PMC9436412; doi:10.7554/eLife.70534)
Supplement: Figure 2—source data 1. [file elife-70534-fig2-data1.docx]

**Figure 2-Source Data 1**

| **RNA** | **N^a^** | **k_association_^b^** |
| --- | --- | --- |
| RNA-10 | 91 | 7.18 ± 2.04 |
| RNA-9a | 95 | 2.90 ± 0.16 |
| RNA-9b | 124 | 2.93 ± 0.23 |
| RNA-8a | 153 | 2.96 ± 0.12 |
| RNA-8b | 77 | 2.32 ± 0.19 |
| RNA-7a | 87 | 1.68 ± 0.07 |
| RNA-7b | 46 | 1.02 ± 0.04 |
| RNA-6a | 44 | 1.57 ± 0.07 |
| RNA-6b | 41 | 0.99 ± 0.07 |
| RNA-4+2 | 85 | 2.95 ± 0.16 |
|  |  | *( x 10^-3^ sec^-1^ )* |

**^a^** *N* represents the number of measured time intervals

**^b^** k_association_ determined for experiments where [RNA] = 10 nM
