# Supplementary material for: Multi-step recognition of potential 5' splice sites by the Saccharomyces cerevisiae U1 snRNP
Source: eLife. 2022 Aug 12;11:e70534. doi: 10.7554/eLife.70534 (PMC9436412; doi:10.7554/eLife.70534)
Supplement: Figure 3—source data 1. [file elife-70534-fig3-data1.docx]

**Figure 3-Source Data 1**

| **RNA** | **N^a^** | **Tau (s)** | **Tau1 (τ_S_ _,_ s)** | **Tau2 (τ_L,_ s)** | **A_L_** | **LLR^b^** |
| --- | --- | --- | --- | --- | --- | --- |
| **RNA-10** | 295 | 179.0 ± 13.1 | 120.5 ± 22.7 | 387.7 ± 97.2 | 0.25 ± 0.16 | *p* < 0.01 |
| **RNA-9a** | 232 | 190.3 ± 14.5 | 136.8 ± 46.1 | 371.3 ± 144.3 | 0.36 ± 0.33 | *p* > 0.1 |
| **RNA-9b** | 273 | 160.3 ± 14.8 | 33.2 ± 5.8 | 292.9 ± 34.2 | 0.49 ± 0.06 | *p* < 0.01 |
| **RNA-8a** | 270 | 75.5 ± 5.9 | 43.1 ± 10.5 | 137.1 ± 45.0 | 0.4 ± 0.19 | *p* < 0.01 |
| **RNA-8b** | 351 | 59.9 ± 3.9 | 48.8 ± 4.2 | 154.0 ± 40.1 | 0.14 ± 0.13 | *p* < 0.01 |
| **RNA-7a** | 132 | 32.7 ± 5.3 | 23.0 ± 2.9 | 175.3 ± 108.7 | 0.11 ± 0.15 | *p* < 0.01 |
| **RNA-7b** | 67 | 36.6 ± 15.0 | 11.3 ± 2.5 | 264.0 ± 177.2 | 0.13 ± 0.08 | *p* < 0.01 |
| **RNA-6a** | 100 | 12.8 ± 1.4 | 12.1 ± 4.8 | 27.4 ± 13.5 | 0.38 ± 0.37 | *p* > 0.1 |
| **RNA-6b** | 59 | 16.7 ± 2.8 | 11.0 ± 2.1 | 36.8 ± 13.9 | 0.32 ± 0.24 | *p* > 0.1 |

**^a^** Number of dwell times combined from replicates for maximum likelihood estimations of single (τ_0_) and double exponential distributions (τ_S_, τ_L_, A_L_).

^b^ Results of a loglikelihood ratio test (LLR) comparing the goodness of fit of single and double exponential estimates evaluated at α = 0.01, where *p* < 0.01 favors a double exponential distribution.
