# Supplementary material for: Multi-step recognition of potential 5' splice sites by the Saccharomyces cerevisiae U1 snRNP
Source: eLife. 2022 Aug 12;11:e70534. doi: 10.7554/eLife.70534 (PMC9436412; doi:10.7554/eLife.70534)
Supplement: Figure 4—source data 1. [file elife-70534-fig4-data1.docx]

**Figure 4—Source Data 1**

| **Mimic** | **RNA** | **N^a^** | **k_off_ (95% CI) ^b^** |
| --- | --- | --- | --- |
| *UU mimic* | RNA-10 | 580 | **7.71** (7.69 7.73) **x 10^-4^ sec^-1^** |
| *UU mimic* | RNA-9a | 235 | **1.40** (1.37 1.43) **x 10^-3^ sec^-1^** |
| *UU mimic* | RNA-9b | 409 | **1.67** (1.64 1.70) **x 10^-3^ sec^-1^** |
| *UU mimic* | RNA-8a | 170 | **1.65** (1.62 1.67) **x 10^-3^ sec^-1^** |
| *UU mimic* | RNA-8b | 126 | **5.83** (5.71 5.95) **x 10^-3^ sec^-1^** |
| *UU mimic* | RNA-7a | 48 | **5.23** (4.86 5.66) **x 10^-3^ sec^-1^** |
| **ΨΨ mimic** | RNA-10 | 550 | **5.50** (5.46 5.54) **x 10^-4^ sec^-1^** |
| **ΨΨ mimic** | RNA-9a | 287 | **10.10** (9.97 10.20) **x 10^-4^ sec^-1^** |
| **ΨΨ mimic** | RNA-9b | 276 | **6.06** (5.97 6.14) **x 10^-4^ sec^-1^** |
| **ΨΨ mimic** | RNA-8a | 172 | **1.33** (1.29 1.36) **x 10^-3^ sec^-1^** |
| **ΨΨ mimic** | RNA-8b | 212 | **4.15** (4.10 4.21) **x 10^-3^ sec^-1^** |
| **ΨΨ mimic** | RNA-7a | 157 | **3.73** (3.57 3.88) **x 10^-3^ sec^-1^** |
| **ΨΨ mimic** | RNA-6a | 67 | **1.28** (1.17 1.39) **x 10^-2^ sec^-1^** |

**^a^** Number of (dwell times) combined from multiple replicates.

**^b^** The calculated k_off_ (and 95% confidence interval for this value) that results from fitting the combined dwell times.
