# Supplementary material for: Multi-step recognition of potential 5' splice sites by the Saccharomyces cerevisiae U1 snRNP
Source: eLife. 2022 Aug 12;11:e70534. doi: 10.7554/eLife.70534 (PMC9436412; doi:10.7554/eLife.70534)
Supplement: Figure 5—source data 1. [file elife-70534-fig5-data1.docx]

**Figure 5-Source Data 1**

| **RNA**  (Mismatch Position) | **N^a^** | **Tau (τ_0_)** | **Tau1 (τ_s_)** | **Tau2 (τ_L_)** | **A_L_** |
| --- | --- | --- | --- | --- | --- |
| **RNA-7a**  (None) | 132 |  | 22.2 ± 2.4 sec | 172.8 ± 100.9 sec | 0.10 ± 0.07 |
| **RNA-6a**  (+6) | 100 | 12.4 ± 1.4 sec | 12.4 ± 1.4 sec |  |  |
| **RNA-6b**  (-1) | 59 | 16.2 ± 2.8 sec | 16.2 ± 2.8 sec |  |  |
| **RNA-1+5**  (+1) | 68 | 23.8 ± 6.3 sec | 23.8 ± 6.3 sec |  |  |
| **RNA-2+4**  (+2) | 45 | 20.3 ± 3.1 sec | 20.3 ± 3.1 sec |  |  |
| **RNA-3+3**  (+3) | 59 |  | 11.4 ± 1.9 sec | 279.2 ± 178.6 sec | 0.21 ± 0.07 |
| **RNA-4+2**  (+4) | 367 |  | 12.5 ± 1.4 sec | 203.5 ± 87.6 sec | 0.09 ± 0.04 |
| **RNA-5+1**  (+5) | 113 |  | 12.6 ± 3.3 sec | 122.8 ± 59.8 sec | 0.14 ± 0.06 |

**^a^** Number of dwell times combined from the replicates and fit to obtain the corresponding parameters.
