# Supplementary material for: Multi-step recognition of potential 5' splice sites by the Saccharomyces cerevisiae U1 snRNP
Source: eLife. 2022 Aug 12;11:e70534. doi: 10.7554/eLife.70534 (PMC9436412; doi:10.7554/eLife.70534)
Supplement: Figure 6—source data 1. [file elife-70534-fig6-data1.docx]

**Figure 6-Source Data 1**

| **RNA** | **Tau (τ_o_, s)^a^** | **Log-likelihood**  **(1 exponential term) ^b^** | **Tau1 (τ_S,_ s) ^c^** | **Tau2  (τ_L, s_)^c^** | **A_S_^c^** | **A_L_^c^** | **Log-likelihood**  **(2 exponential terms)^d^** |
| --- | --- | --- | --- | --- | --- | --- | --- |
| **RNA-2+7 - A(+1)** | 7.5 ± 0.5 | -1563.0 | 6.9 ± 0.2 | 92.0 ± 42.4 | 0.99 ±0.01 | 0.01 | -1543.3 |
| **RNA-2+7 - C(+1)** | 13.8 ± 2.8 | -322.3 | 8.6 ± 1.1 | 72.2 ± 33.2 | 0.90 ± 0.07 | 0.10 | -308.9 |
| **RNA-2+7 - U(+1)** | 12.1 ± 3.7 | -1563.0 | 7.1 ± 0.6 | 188.1 ± 68.1 | 0.97 ± 0.02 | 0.03 | -284.3 |

**^a^** Fit parameter to an equation containing a single exponential term.

**^b^** Log-likelihood output for the fit to an equation with a single exponential term.

**^c^** Fit parameters to an equation containing two exponential terms. A_S_ is the amplitude of the first exponential term, τ_S_. A_L_ is the amplitude of the second exponential term, τ_L._

**^d^** Log-likelihood output for the fit to an equation with two exponential terms. The more positive log-likelihood indicates these data are better fit to equations containing two terms.
